# Bilateral Inferior Altitudinal Visual Field Defect in Recurrent Intracranial Meningioma: A Case Report

**DOI:** 10.7759/cureus.4436

**Published:** 2019-04-11

**Authors:** Ahmad Kamal Ghanimi Zamli, Tan Chew-Ean, Wan Hazabbah Wan Hitam

**Affiliations:** 1 Ophthalmology, Universiti Sains Malaysia, Kota Bharu, MYS

**Keywords:** meningioma, bilateral inferior altitudinal hemianopsia

## Abstract

Altitudinal visual field defect is a rare presentation of retrochiasmal lesion especially when bilateral visual fields were affected. In fact, bilateral inferior altitudinal visual field defect (BIAVFD) usually occurred in patients who survived a gunshot injury to the occipital lobe or as a direct trauma to the brain. We report a rare case of BIAVFD secondary to occipital meningioma. A high index of suspicion enables timely investigation and diagnosis when dealing with atypical presentation of intracranial meningioma.

## Introduction

Meningioma is a primary intracranial neoplasm that arises from the meningothelial cells of the arachnoid layer which surrounds the spinal cord and the brain [1]. The most common location for meningioma to arise is within the cerebellar meninges that accounts for almost 90% of the cases [2]. Patients with meningioma usually demonstrate symptoms of generalized headache, dizziness, seizure, deterioration of cognitive and motor function, and visual disturbances [3].

Bilateral inferior altitudinal visual field defect (BIAVFD) or inferior hemianopsia is a rare presentation of a retrochiasmal pathology. BIAVFD is mainly associated with nonarteritic anterior ischemic optic neuropathy (NAAION), retinal lesion (choroiditis and coloboma) or optic nerve lesion (glaucoma, optic disc drusen and optic nerve hypoplasia) in which all of these mainly affect structures anterior to the optic chiasm [4,5]. Nonetheless, the current report involved a case in which we observed a patient with occipital meningioma presenting with bilateral inferior altitudinal hemianopsia.

## Case presentation

A 52-year-old gentleman presented with generalized headache, blurring of vision and unsteady gait for one-year duration. He described his headache was throbbing in nature and progressed to be persistent throughout the day. There was no aura and no preceding history of seizure. He also started to have unsteady gait, followed by blurring of vision especially at downgaze.

Otherwise, he had no known underlying medical illness. He denied of any history of head trauma or fall. There was no memory loss or personality changes. He was recently diagnosed to have ischemic heart disease via stress test.

At presentation, he was alert and conscious with stable vital signs. Visual acuity was 6/6 on both eyes. Optic nerve function tests were normal with no relative apparent pupillary defect. The anterior and posterior segment examination was unremarkable. Surprisingly, both optic disc appeared normal with cup-disc ratio of 0.3, and no signs of papilloedema or optic nerve atrophy seen. Extraocular muscles movement were full in all directions. The only positive finding was the confrontational test revealed a left lower homonymous quadrantanopia. Systemic review and neurological examination were normal.

**Figure 1:**
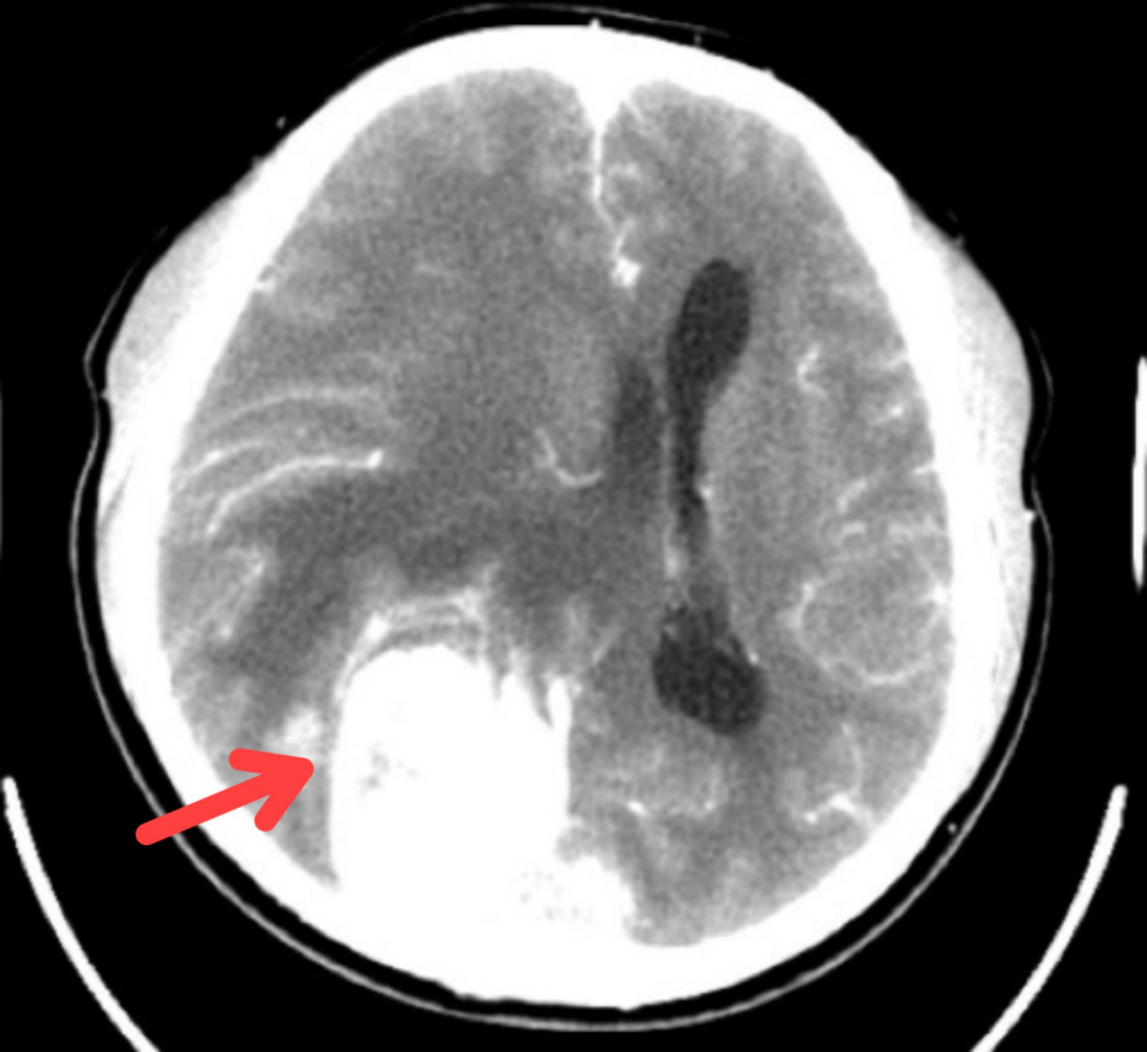
Computed tomography scan with contrast during first presentation showed a huge right occipital lobe tumour with mass effect.

**Figure 2:**
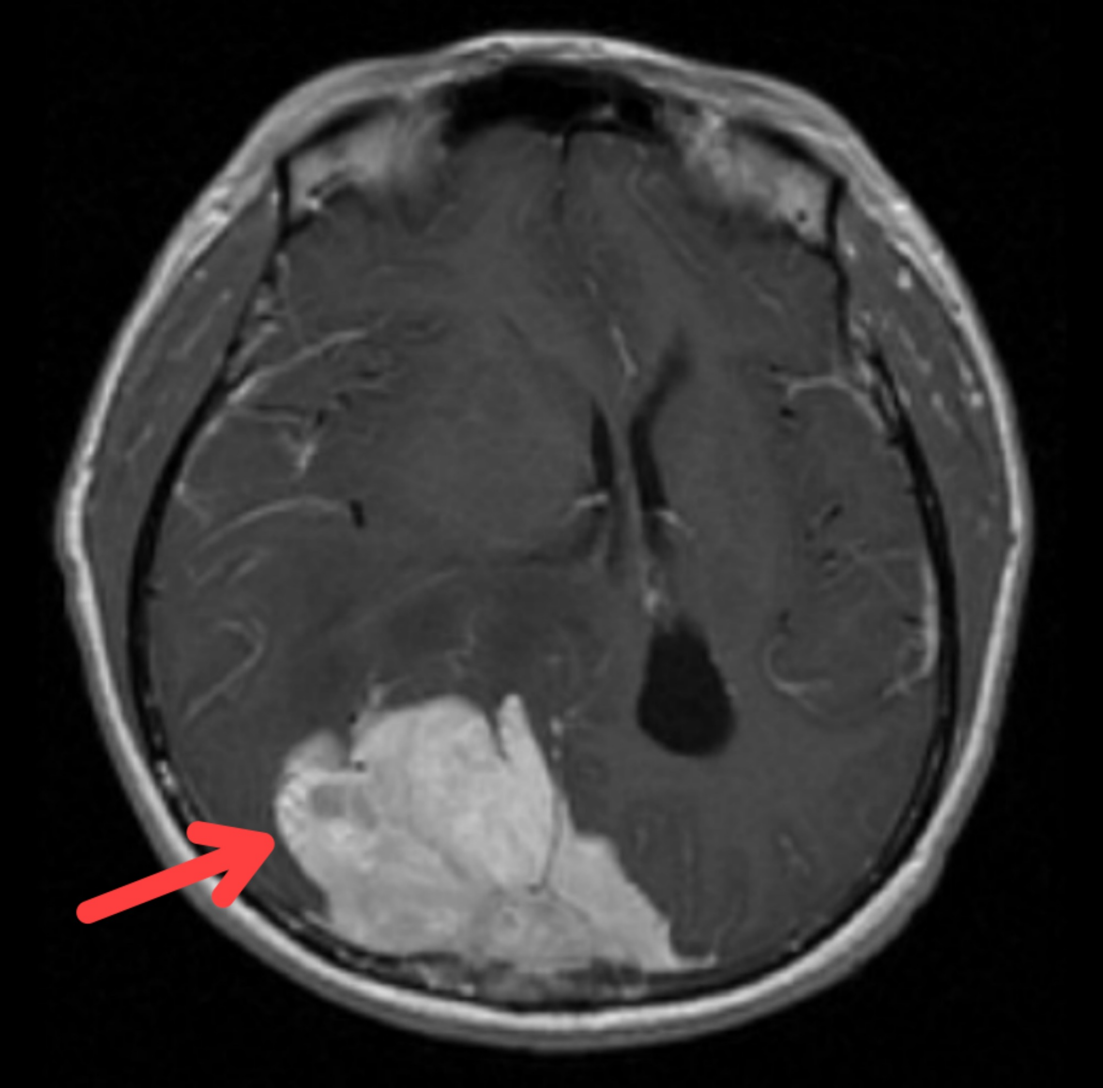
Magnetic resonance imaging showed a huge right occipital lobe tumour with mass effect.

He underwent computed tomography (CT) scan and whereby he was noted to have a large solitary tumour located at the parasagittal area of occipital region suggestive of parasagittal meningioma (Figure 1). Magnetic resonance imaging (MRI) showed similar findings (Figure 2). He was then referred to neurosurgical team and tumour excision was done. Post operatively, he recovered fully with no sequalae, but his visual field remained the same. Histopathological examination of the tumour excised was confirmed to be transitional meningioma.

**Figure 3:**
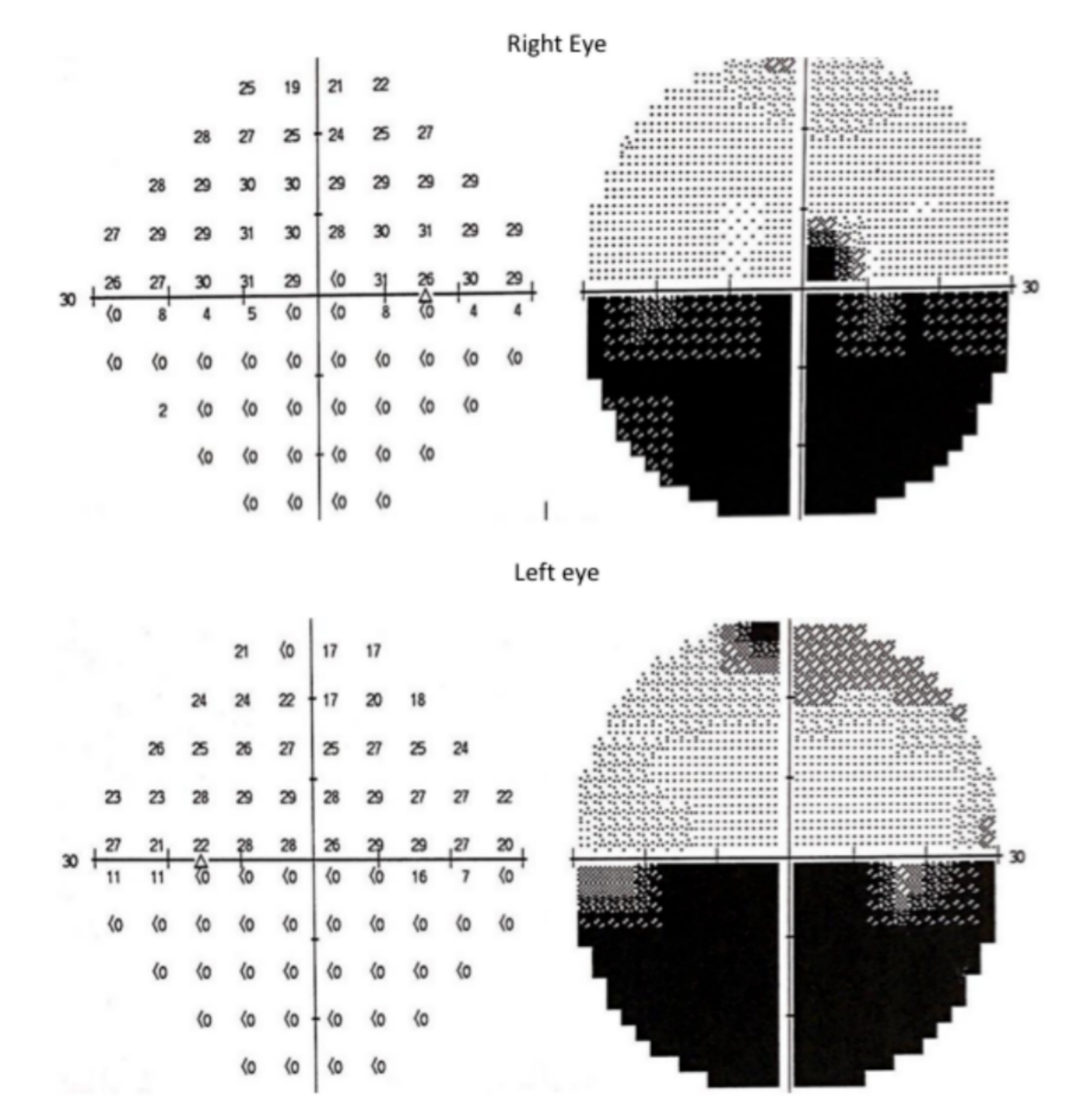
Humphrey visual field showed bilateral inferior altitudinal visual field defect.

Surveillance MRI after one year of post-surgery revealed a residual tumour at the right occipital area with another new tumour growth at the left occipital area. He was then subjected to radiotherapy treatment. However, a second craniotomy with excision of tumour surgery was performed at the second year of follow-up after radiotherapy failed to shrink the tumour. Prior to his second surgery, his visual field started to deteriorate whereby he was unable to gauge the downgoing staircases (Figure 3).

During his ophthalmological follow-up, his visual acuity was still good with 6/7.5 over both eyes. Humphrey visual field demonstrated a bilateral altitudinal visual field defect which was denser at the left inferior quadrant. Fundus examination had no sign of optic atrophy. Currently, the patient is under ophthalmology regular follow-up and on-going visual rehabilitation therapy.

## Discussion

Meningioma presents with an incidence rate of 8.33 per 100,000 populations in the United States, which is four times higher than the incidence rate of all the brain and other nervous systems tumors in Malaysia (1.8 for male and 1.7 for female per 100,000 populations) [2,6]. Meningioma is commonly seen in female by the ratio of 2:1 and it mainly affects the elderly [2]. Majority of meningioma are benign solitary tumour, but vary in location and size, which determine the surgical options and post-operative complications [1,2]. Furthermore, these factors also have led to the diversity of clinical manifestations and masked the disease's severity until the presentation was late, similar to our case report.

Even though meningioma has a high survival rate (81.5% survival rate in 10 years), patients may develop sequalae either due to its mass effect or as a manifestation of post-operative surgical complications [2]. In a study by Liouta et al., intracranial meningioma may also exert neurocognitive deterioration in which many of their subjects developed a reduction in scores in cognitive function such as memory, attention, visuospatial function and fine motor performance [3]. However, the most debilitating sequelae that can occur is when the visual function is impaired. In such condition, not only the patients’ quality of life is affected, but they may also succumb to major depressive disorder and suicidal ideation.

Many reported incidents of visual field problem in meningioma usually found in cases of tuberculum sellae meningioma. In these cases, patients usually presented with bitemporal hemianopia, bilateral superior quadrantanopia or unilateral visual loss [7, 8]. Such presentation occurred due to its close proximity to the optic chiasm and optic nerve tract. In fact, most of meningiomas that arise form sellar and parasellar region will almost be associated with not only visual field defects but also cranial nerve palsies, proptosis, hydrocephalus or in combinations [7]. Other visual fields defects in meningioma cases are considered to be rare especially for patients with BIAVFD.

BIAVFD is one of the presentations of occipital lobe lesion, which can be due to penetrating injury such as a bullet or as direct trauma to this area. Such presentation occurs when there is a direct insult towards bilateral occipital lobe which lies above the calcarine fissure. Should the insults affect the area inferior to the calcarine fissure, superior field defect resulted, and almost inevitably a fatal condition due to its close proximity to the cerebral sinuses [5,9]. This type of visual field defect may also be caused by cerebrovascular accidents of the posterior circulation, which supplies the inferior portion of the occipital lobe. Contrary to this, BIAVFD in our patient occurred due to the recurrent meningioma that involved bilateral occipital lobe superior to the calcarine fissure.

In our patient, the progression of the diseases was slow but severe, and due to this, his quality of life was affected even though he has 6/6 vision at both eyes. In patients with BIAVFD, this is an expected situation faced by those bearing this disease as they will have difficulty in reading, climbing stairs or even estimating distance while walking. Frequent stumbling and falling in elderly patients will be dangerous and this can lead to fractures and morbidity.

Previously, the management of BIAVFD was mainly conservative and the patient has to bear the visual field defect and limit his daily life activities. With the advent of visual rehabilitation, Romano has suggested two approaches for management of visual field defect in patients. First is by implementing compensatory strategies such as prisms or saccadic training which can improve the ability to scan object within a visual field defect [10]. Even though this strategy was believed to improve reading speed, it has little benefit in patients with BIAVFD and might be dangerous especially when implementing prism [11].

While the second strategy is by restitution therapy in which such techniques can enlarge the visual field by using a home-based vision restoration therapy (VRT). This VRT stimulates the border zone between the seeing and the blind field repetitively on a daily basis over many months [10]. By stimulating the retina, this rehabilitation therapy has improved up to 5.0° in central vision for retrochiasmal pathology [12]. Such excellent improvement can definitely improve patients’ daily life activities and avoidance of injury due to visual field defects [13].

## Conclusions

In retrochiasmal lesion, BIAVFD can be atypical manifestation of occipital meningioma when both of the occipital lobes were affected. High index of suspicion with timely diagnosis and prompt treatment is the best management for better survival outcome. It is incumbent for all team that facilitates the management of this patient to offer rehabilitative measures to patients that have any form of visual field defect.
